# Building a pre-surgical multiparametric-MRI-based morphologic, qualitative, semiquantitative, first and high-order radiomic predictive treatment response model for undifferentiated pleomorphic sarcoma to replace RECIST

**DOI:** 10.1186/s40644-025-00873-1

**Published:** 2025-04-26

**Authors:** Raul F. Valenzuela, Elvis Duran-Sierra, Mathew Antony, Behrang Amini, Sam Lo, Keila E. Torres, Robert S. Benjamin, Jingfei Ma, Ken-Pin Hwang, R. Jason Stafford, Dejka Araujo, Andrew J. Bishop, Ravin Ratan, Wei-Lien Wang, Jossue Espinoza, Pia V. Valenzuela, Chengyue Wu, John E. Madewell, William A. Murphy, Colleen M. Costelloe

**Affiliations:** 1 The University of Texas MD Anderson Cancer Center, Houston, United States; 2 Texas A and M University, College Station, United States

**Keywords:** Soft tissue sarcoma (STS), Undifferentiated pleomorphic sarcoma (UPS), Pathology-assessed treatment effect (PATE), Multiparametric MRI, Radiomics

## Abstract

**Background:**

Undifferentiated pleomorphic sarcoma (UPS) is the largest subgroup of soft-tissue sarcomas. It demonstrates post-therapeutic hemosiderin deposition, granulation tissue formation, fibrosis, and calcification. Our research aims to establish the multiparametric MRI (mp-MRI) value for predicting UPS treatment response.

**Methods:**

An IRB-approved retrospective study included 33 extremity UPS patients with pre-operative mp-MRI, including diffusion-weighted imaging (DWI), contrast-enhanced susceptibility-weighted imaging (CE-SWI), and perfusion-weighted imaging with dynamic contrast-enhancement (PWI/DCE), and surgical resection between February 2021 and May 2023. Lesions were visually classified on CE-SWI into one of 6 morphology patterns. On PWI/DCE, lesions were classified into one of 6 patterns, and time-intensity curves (TICs) were classified as types I-V. Patients were categorized into three groups based on the percentage of pathology-assessed treatment effect (PATE) in the surgical specimen: Responders (> = 90% PATE, *n* = 16), partial-responders (31–89% PATE, *n* = 10), and non-responders (< = 30% PATE, *n* = 7).

**Results:**

At post-radiation therapy (PRT), a CE-SWI Complete-Ring pattern was observed in 71% of responders (*p* = 7.71 × 10^–6^). On PWI/DCE images, 79% of responders displayed a Capsular pattern (*p* = 1.49 × 10^–7^), and 100% demonstrated a TIC-type II (*p* = 8.32 × 10^–7^). ROC analysis comparing responders (*n* = 14) vs. partial/non-responders (*n* = 16) at PRT showed that the model combining PWI/DCE TIC-type II, PWI/DCE Capsular pattern, and CE-SWI Complete-Ring pattern yielded the highest classification performance (AUC = 0.99), outperforming PWI/DCE Capsular + TIC-type II (AUC = 0.97), PWI/DCE Capsular (AUC = 0.89), PWI/DCE TIC-type II (AUC = 0.88), and CE-SWI Complete Ring (AUC = 0.79). Contrary to prior reports, DWI/ADC played a secondary role in predicting response: ADC mean & skewness (AUC = 0.63). RECIST demonstrated 100% stability at PRT and 100% pseudo-progression at PC in responders and partial/non-responders (AUC = 0.47).

**Conclusion:**

Mp-MRI-derived features are valuable in assessing UPS treatment response. A pre-operative model that combines PWI/DCE TIC-type II, PWI/DCE Capsular pattern, and CE-SWI Complete Ring pattern can reliably predict successfully treated UPS with > = 90% PATE, outperforming RECIST, which was proven unreliable in separating responders from partial/non-responders. Institutions that have not yet implemented CE-SWI can rely on a single-sequence approach based on PWI/DCE, combining the presence of TIC II and Capsular enhancement as criteria for response prediction.

## Introduction

Undifferentiated pleomorphic sarcoma (UPS) is the largest soft-tissue sarcoma (STS) subgroup, representing approximately 20% of all cases. UPS is considered a diagnosis of exclusion when all identifiable lines of histopathologic differentiation have been excluded. It can arise anywhere in the body and any age group, although it is more common in older patients [1, 2]. UPS represents an archetypical cellular STS imaging tumor model significantly different from primary myxoid, chondroid, lipomatous, or fibrous-rich STS [2, 3].

The Response Evaluation Criteria in Solid Tumors (RECIST) version 1.1 [4] is mainly based on the size measurement of lesions. Using RECIST for treatment response assessment provides standardized lesion size metrics that enable interobserver reliability and facilitate data comparison from different trials [5]. Nevertheless, RECIST is relatively insensitive to detecting early tumor response and incapable of characterizing lesion composition, viability, and tumor-associated inflammation or immune cell infiltration [5]. After treatment, UPS shows reduced cellularity with degenerative cytological changes, necrosis, and hyalinization. Hemorrhage, hemosiderin deposition, and foamy macrophages can also be observed. These findings help determine the percentage of pathology-assessed treatment effect (PATE) [6, 7]. A high percentage of PATE is a strong indicator of a favorable prognosis in sarcomas [7]. Increased histologic necrosis after neoadjuvant therapy may lead to higher rates of R0 resection and facilitate limb salvage in tumors deemed unresectable [7, 8]. STS patients often receive systemic treatment with anthracyclines and alkylating agents. In our institution, systemic therapy most commonly includes doxorubicin and ifosfamide, frequently preceding radiation therapy, typically before surgery.

### Advantages of multiparametric MRI (mp-MRI)

Traditional methods of assessing tumor response, such as RECIST, the World Health Organization (WHO) criteria, and volumetric measurements, have limitations in accurately evaluating tumor response [1, 4]. Typically based on conventional imaging, these methods focus on the tumor size and morphology. UPS generally appears as a heterogeneously enhancing mass in the soft tissues and displays a similar signal to muscle on T1-weighted sequences, representing a diagnostic challenge. On T2-weighted sequences, central hyperintense regions can indicate necrosis or hemorrhage and could lead to a misdiagnosis of a benign hematoma [9]. To overcome such limitations, mp-MRI, including diffusion-weighted imaging (DWI) and perfusion-weighted imaging with dynamic contrast-enhanced imaging (PWI/DCE), provides a more comprehensive assessment of the tumor's biology, including its cellularity and vascularity [10]. Susceptibility-weighted imaging (SWI) is sensitized to T2*-related signal contrast, adding valuable information about hemorrhage, fibrosis, and calcifications [11]. Hence, mp-MRI can help identify post-therapeutic histologic changes, such as the development of granulation tissue and fibrosis, which may be difficult to distinguish from viable tumor tissue using conventional imaging methods [5, 12].

Diffusion-weighted imaging and apparent diffusion coefficient (DWI/ADC) are sensitive to the density of tumor cells within soft tissue sarcomas (STS). DWI utilizes the random movement of water molecules to characterize the cellularity of malignant tumors. Malignant tumors generally have fewer extracellular spaces and less cytoplasm than benign tissues [13–15]. The apparent diffusion coefficient (ADC) values can be computed using either a simple mono-exponential model or more sophisticated models to quantify the degree of diffusion [15, 16]. Malignancies typically exhibit restricted diffusion, resulting in lower ADC values, which helps to distinguish them from benign lesions that usually demonstrate higher ADC values. Effective therapy leading to cell death increases water diffusion and results in higher ADC values. DWI/ADC has shown value as a potential biomarker of response in sarcoma patients undergoing neo-adjuvant therapy (NAT) [17–19].

Perfusion-weighted imaging with dynamic contrast enhancement (PWI/DCE) acquires a series of rapid scans after injecting an intravenous contrast agent. It allows for the analysis of tissue perfusion kinetics [3] by qualitative, semi-quantitative, or quantitative [3, 10] approaches. It helps identify viable malignant tumor tissue by showing distinct perfusion patterns. Malignant tissues usually show rapid early enhancement compared to benign tumors [12, 20]. PWI/DCE can assist in guiding biopsies and identifying histologic changes after therapy. When combined with DWI, PWI/DCE increases the accuracy of diagnosing therapeutic responses compared to conventional, anatomic post-contrast imaging alone [3, 5].

Susceptibility-weighted imaging (SWI) is a 3D high-spatial-resolution, velocity-corrected gradient-echo MRI sequence. It creates images with magnitude and filtered-phase information [11] and uses tissue magnetic susceptibility differences to generate signal contrast. The signal arises from paramagnetic (hemosiderin), diamagnetic (minerals and calcifications), and ferromagnetic (metal) molecules, resulting in a loss of signal [11, 21] that can be analyzed for patterns. Contrast-enhanced susceptibility-weighted imaging (CE-SWI) can simultaneously detect the hemorrhage-related T2* susceptibility effect, the necrotic fluid T2 signal, and contrast-induced T1 shortening from the viable enhancing tumor [22, 23]. Recent studies have shown that CE-SWI can differentiate viable tissue from hemorrhagic/necrotic components in soft tissue sarcomas, potentially providing valuable biomarkers for tumor treatment response assessment [22, 23].

An mp-MRI-based model capable of replacing RECIST for local tumor evaluation can contribute to a more accurate prediction of UPS treatment response. This could help reduce patients’ unnecessary treatment toxicity and increased risk for resistance to future chemotherapeutic agents and allow them to transition to surgery sooner and with potentially improved outcomes. This could enable building a theranostic/predictive model, allowing therapy modifications or adjustments during NAT.

### Study objective

Following a 3-year effort to separately validate the use of DWI/ADC, PWI/DCE, and CE-SWI in predicting UPS treatment response [19, 24], we now aim to determine the best predictive model and its clinical relevance and utility by comparing the performance of multiparametric MRI-derived morphologic, qualitative, semiquantitative, first- and high-order radiomics features. Analysis of this data will be used to identify the highest-performing combination of parameters to create a clinically usable model that can reliably predict PATE and overall therapeutic effectiveness, potentially replacing RECIST for local tumor evaluation. This study represents the first demonstration of mp-MRI feature-based predictive modeling, including DWI, PWI/DCE, and CE-SWI, for extremity UPS treatment response assessment, which could potentially be generalized to other types of STS.

## Methods

### Disclosures and statements

#### Ethics and methodology

The current research article does not include animal or human experimentation and is not part of a clinical trial. All included authors have contributed to the current article and all data collection, management, and processing methods were performed by all the institutional and generally accepted relevant clinical, research and ethical guidelines and regulations, including but not limited to the protection of data veracity and personal health information (PHI).

#### IRB and waiver of consent

Due to the study's retrospective nature, The UT MD Anderson Cancer Center Institutional Review Board waived the need to obtain informed consent and gave ethical and administrative approval to our research under the IRB identifier PA16 - 0857 Protocol Name: “Utility of imaging of bone and soft tissue tumors and disease and treatment-related changes for diagnosis, prognosis, treatment response, and outcome.”

#### Data availability

The datasets used and analyzed during the current study are available from the corresponding author upon reasonable request.

#### Funding

Charitable funding sources of our research include The John S. Dunn, Sr. Distinguished Chair in Diagnostic Imaging, and the M.R. Evelyn Hudson Foundation Endowed Professorship funds.

#### Institutional MRI protocol

We performed functional MRI sequences, including DWI/ADC, CE-SWI, and PWI/DCE [3, 5]. Parameters were tailored according to MRI vendor and field strength. During the pre-operative treatment, multiple scans were acquired for each patient and compiled into three time points: Baseline (BL, pre-therapy), post-systemic chemotherapy (PC), and pre-operative/post-radiation (PRT) time points. For patients with STS, we typically conduct a pre-therapy baseline study, one to three MRIs during systemic chemotherapy, and at least one post-radiation study one to two months after radiation therapy and immediately before surgical resection.

#### Patient population, patient inclusion, and exclusion

This retrospective study analyzed a total of 5,135 multiparametric MRI (mp-MRI) scans performed for extremity soft tissue sarcomas (STS) using our institutional fleet of 29 magnets, which includes both 1.5 T and 3.0 T scanners from two different manufacturers, between February 2021 and May 2023. During this period, 643 UPS mp-MRI studies were completed, encompassing pre-operative assessments of primary tumors and post-operative surveillance studies. We excluded all myxoid-UPS and post-operative surveillance cases. We focused our study on 33 surgically resected cases of undifferentiated pleomorphic sarcomas (UPS) that had undergone presurgical mp-MRI. The following cases were excluded from the analysis:

- 18 cases that did not receive neoadjuvant chemotherapy before surgical resection (excluded from the PC analysis only).
- 3 cases that did not receive neoadjuvant radiotherapy before surgical resection (excluded from the PRT analysis only).
- One case did not include post-radiotherapy contrast-enhanced susceptibility-weighted imaging (CE-SWI) (excluded only from the CE-SWI PRT analysis).

The study population of 33 patients ranged in age from 36 to 85 (the average age was 64, Table 1). Twenty were male (61%), and 13 were female (39%).

**Table 1:** Summary of the UPS patient population included in the study

Total UPS Patients	33
Total Male	20 (61%)
Total Female	13 (39%)
Average Age	64 years (range 36–85 years)
Responders (≥ = 90% PATE)	16
Partial Responders (Between 89 and 31% PATE)	10
Non-Responders (≤ 30% PATE)	7
Total External Baseline (Conventional Imaging)	10
Total CE-SWI Baseline Studies	23
Total PWI/DCE Baseline Studies	23
Total DWI/ADC Baseline Studies	23
Total CE-SWI Post-Chemo Studies	15
Total PWI/DCE Post-Chemo Studies	14
Total DWI/ADC Post-Chemo Studies	15
Total CE-SWI Post-Radiation Studies	29
Total PWI/DCE Post-Radiation Studies	30
Total DWI/ADC Post-Radiation Studies	30

#### Surgical specimen pathology assessment

Following our institution's standard of practice reporting pathology treatment effects, patients were categorized into three groups based on the surgical specimen's pathology-assessed treatment effect (PATE) percentage. Tumors demonstrating over 90% PATE were classified as responders (R, *n* = 16), tumors with a PATE in the 31–89% range were labeled as partial responders (PR, *n* = 10), and tumors with a PATE of 30% or less were considered non-responders (NR, *n* = 7).

#### MRI exams

Of the 33 patients, 10 underwent BL MRI studies outside of our institution without mp-MRI, while their subsequent PC and PRT mp-MRIs were obtained at our institution. These 10 BL patients were excluded from the multiparametric analysis although included in the conventional size-based RECIST, WHO, and volume analysis. For the remaining 23 patients, a complete set of advanced MRI studies, including PC and PRT, was performed at our institution. 23 patients were included in the BL group, 15 in the PC group, and 30 in the PRT group (Table 1).

#### MRI storage and post-processing

MRI data sets were transferred to the institutional Picture Archiving and Communication System (IntelliSpace PACS, Philips, Amsterdam, Netherlands). MR images were retrieved from the institutional database, and each tumor was manually contoured in three dimensions, creating a tumor Volume of Interest (VOI) segmentation. MIM software version 7.1.4 (MIM Software Inc., Cleveland, USA) was used to outline, process, and generate VOIs from mp-MRI. VOI segmentation provided total tumor representation, including all areas of necrosis and enhancing tumor, allowing a more reliable tumor mapping than an arbitrary 2-D region of interest (ROI) segmentation. Although manual segmentation is time-consuming and requires expert Research Assistant (RA) contouring and oversight by a senior radiologist, it is our preferred method. No alternative effective automated segmentation method is currently available in our institution. This manual 3-D tumor segmentation process took approximately 60 min per patient. The segmented tumor VOI files were subsequently exported from MIM as RT-Struct files for further analysis using an in-house developed Python-based vendor-and sequence-neutral application: Cancer Radiomic and Perfusion Imaging (CARPI) automated framework [17], capable of intensity histogram-based first-and high-order radiomic feature extraction from advanced MRI sequences. A total of 107 radiomic features were extracted from the CE-SWI and DWI/ADC tumor VOIs, including shape (14 features), first-order statistics (18 features), and texture (75 features). Specific details on the 107 volumetric radiomic features extracted by CARPI have been previously presented [17]. CARPI also extracted semi-quantitative PWI/DCE parameters from time-intensity curves (TICs): Wash-in rate (WiR), wash-out-rate (WoR), peak enhancement (PE), wash-in area under the curve (WiAUC), time-to-peak (TTP), wash-out area under the curve (WoAUC), and total area under the curve (AUC) [17, 25]**.** Tumor size for all three orthogonal planes was registered for all time points and used to estimate RECIST, WHO, and volume data.

#### Conventional size-based response assessment metrics

Maximum diameter (RECIST), multiplication of the longest and perpendicular cross-sectional diameters (WHO), and volumetric measurements were measured for all 33 patients at PC and PRT with respect to BL, comparing responders and partial/non-responders. RECIST, WHO, and volume criteria for partial response (PR) threshold were set at 30, 50, and 50% decrease, respectively. Progressive disease (PD) threshold was set at 20, 25, and 25% increase, respectively [26, 27]. 

#### PWI/DCE morphologic patterns and TICs

PWI/DCE images observed at PRT were categorized into six groups: Capsular, Unipolar, Bipolar, Non-Nodular, Semi-Lunar, and Solid Enhancement (Table 2). In PWI/DCE, the TIC usually displays three stages of perfusion: 1) upslope, which reflects contrast wash-in; 2) plateau, which represents the steady state of contrast within the interstitial fluid but may not be visible in all lesions; and 3) downslope, which reflects contrast wash-out as gadolinium passes out of the tissues under examination. The TIC shape was subjectively assigned to one of five curve types based on their morphology by an experienced radiologist: Types I, II, III, IV, and V (Table 3). Lesions were categorized into TIC III, IV, and V when displaying a rapid wash-in/early upstroke curve [5, 10].

**Table 2:**
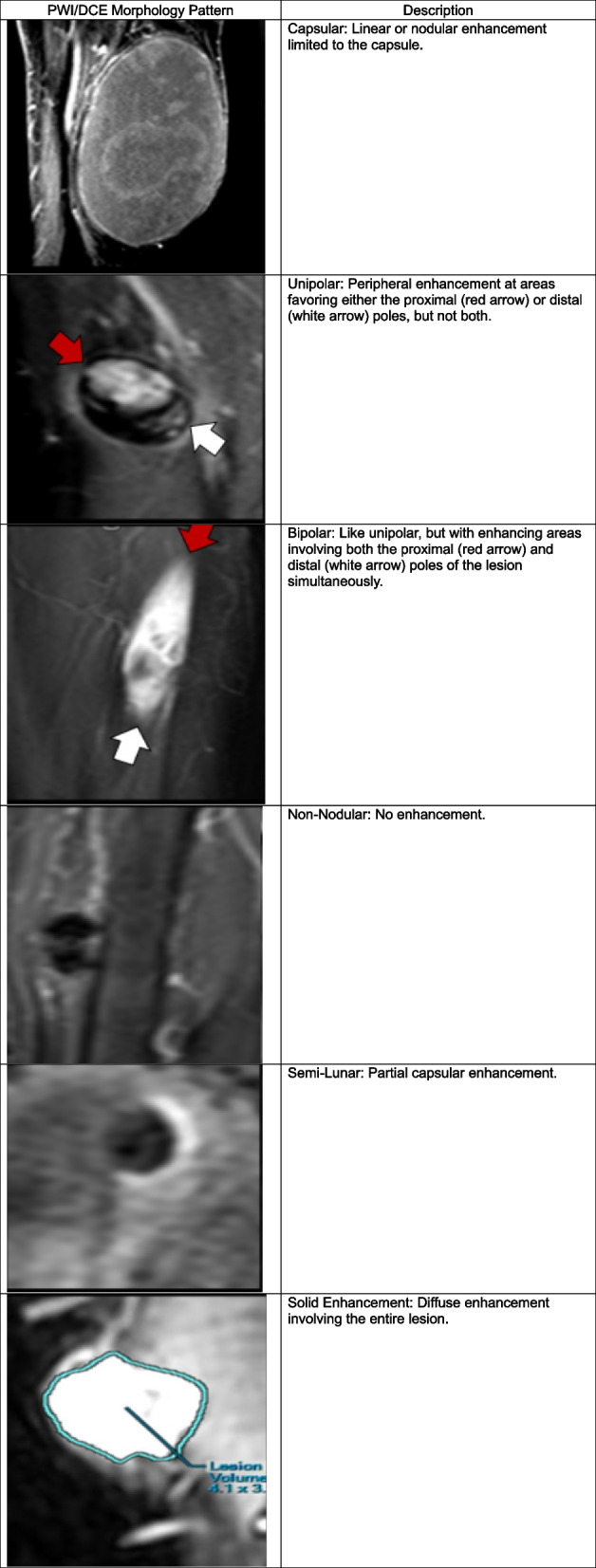
PWI/DCE morphologic pattern definitions

**Table 3:**
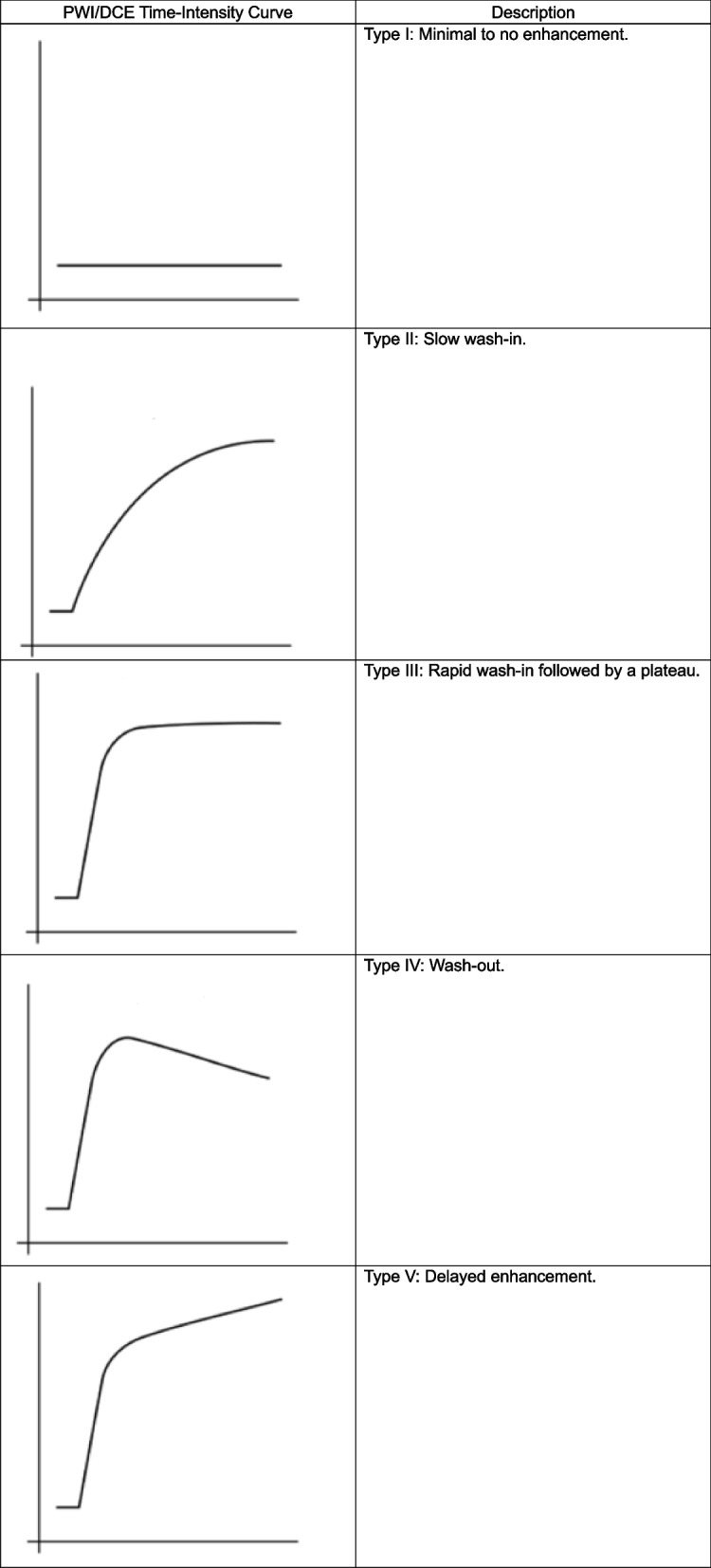
PWI/DCE time-intensity curve types

#### CE-SWI morphologic patterns

CE-SWI images were categorized into six T2* morphological patterns observed at PRT as Complete-Ring, Full-Blooming, Globular, Incomplete-Ring, Interstitial, and No-Blooming (Table 4) [22, 23]. These morphological patterns were compared among R, PR, and NR to determine each category's most frequently recurring patterns.

**Table 4:**
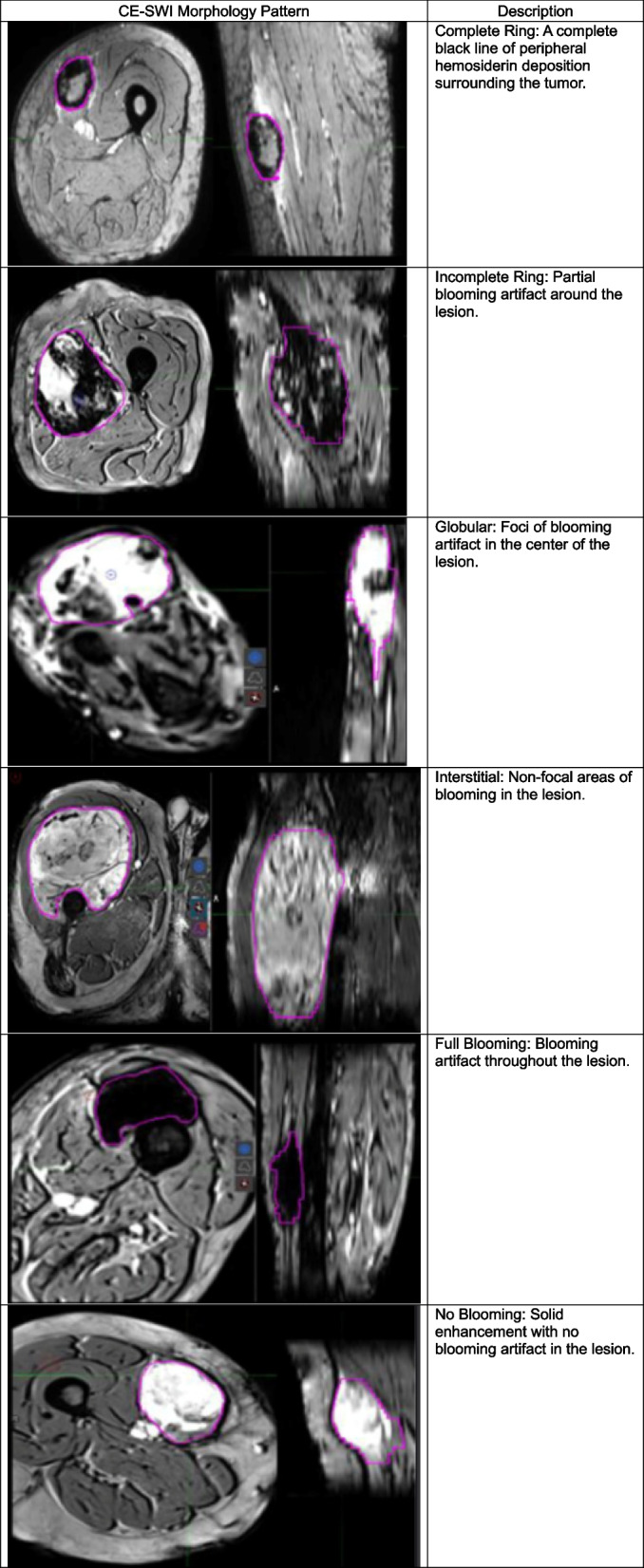
CE-SWI morphologic pattern definitions

#### Statistical analysis

One-way chi-squared tests were performed to assess the association of CE-SWI morphologic patterns, TIC curves, and PWI/DCE morphologic patterns with response. The radiomic features and semi-quantitative variables were compared in responders vs. partial/non-responders using two-tailed non-parametric Wilcoxon rank-sum tests. A receiver operating characteristic (ROC) analysis of the most relevant mp-MRI features was performed for discriminating responders from partial/non-responders. All statistical analyses were implemented in Python 3.10.13 using the SciPy library version 1.12.0 and the Scikit-Learn library version 1.4.1. Statistical significance was assessed at 5% (*P* < 0.05).

## Results

### RECIST, WHO, and volume metrics

**Fig. 1:**
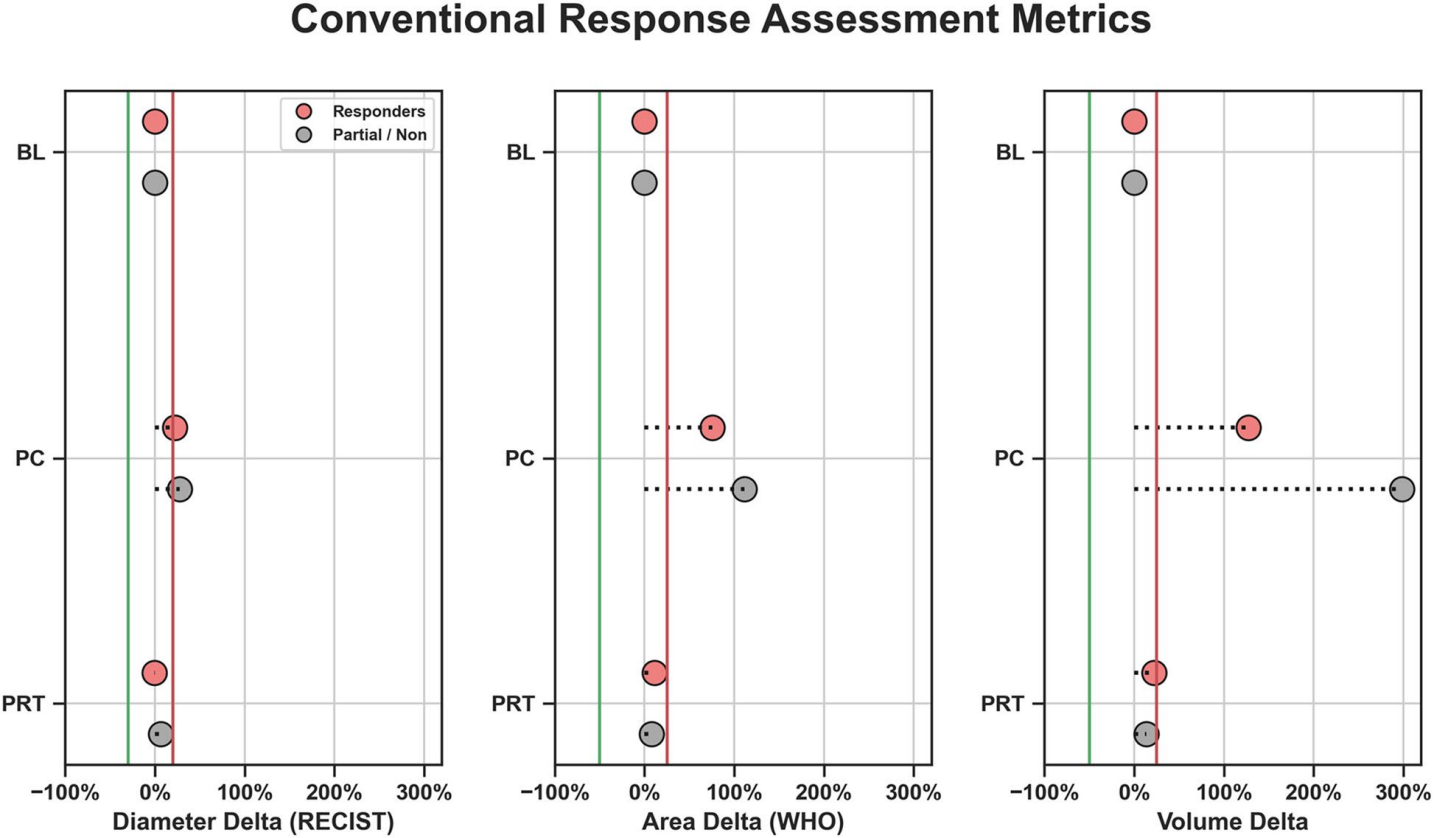
Conventional sized-based response assessment metrics in responders and partial/non-responders comparing changes in diameter (RECIST), multiplication of the longest and perpendicular cross-sectional diameters (WHO), and volume at post-chemo (PC) and post-radiation (PRT) with respect to baseline (BL). The green and red vertical lines indicate partial response and progression thresholds, respectively, for RECIST, WHO, and volume. RECIST, WHO and Volume analysis all show universal pseudo progression at PC and stability at PRT, rendering these size-based metrics useless to separate R vs PR/NR

All patients displayed size changes at the post-radiation time point that when compared to their respective BL, fell within the range of stability, namely between + 20% and − 30% for RECIST and + 25% and − 50% for WHO and volume (Fig. 1) [26]. Pseudo-progression occurs when a tumor increases in size from its original baseline, followed by a reduction in size on subsequent imaging or pathological assessment, without any change in therapy or indication of progression [5, 28]. All PR/NR cases demonstrated pseudo-progression at the end of systemic treatment, crossing the threshold of + 20% for RECIST and + 25% for WHO and volume assessments. All R presented WHO and volumetric pseudo-progression at PC (Fig. 1).

### DWI/ADC radiomics at PRT

**Fig. 2:**
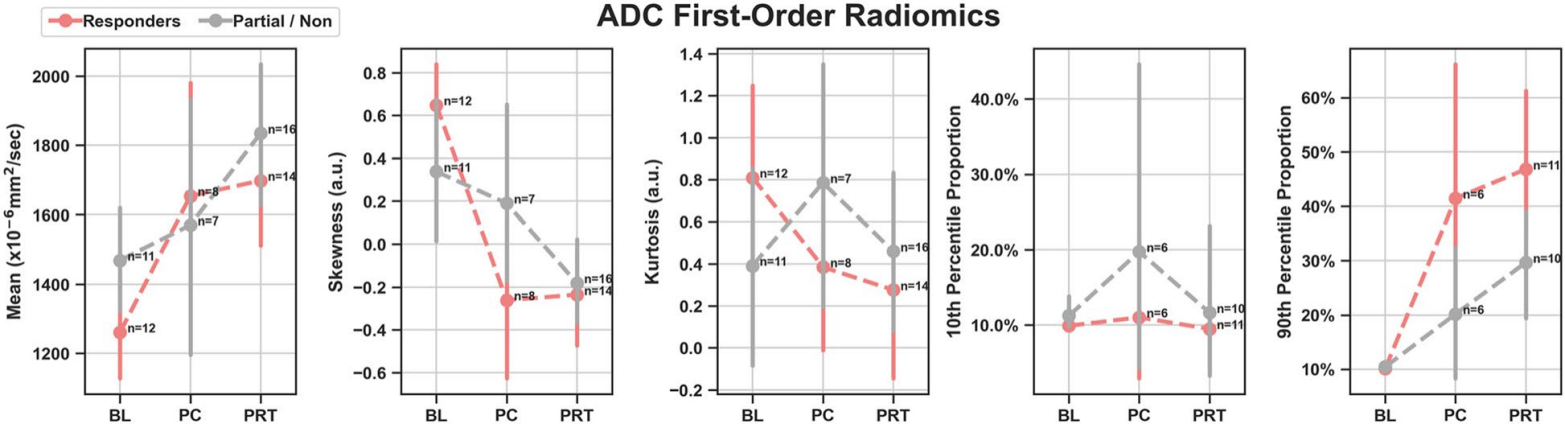
Scatter plots showing means with 95% confidence intervals of ADC first-order radiomics at baseline (BL) vs. post-chemo (PC) vs. post-radiation (PRT) in responders and partial/non-responders. a.u.: Arbitrary Units. ADC mean and ADC skewness demonstrated the highest performance discriminating R vs PR/NR

First-order radiomics: Responders displayed a 35% increase in ADC mean (*P* = 0.0034), a 136% decrease in skewness (*P* = 0.0001), and a 363% increase in 90 th percentile proportion (*P* = 0.0009) with respect to BL (Fig. 2). Partial/non-responders displayed a 25% increase in ADC mean (*P* = 0.0136), 154% decrease in skewness (*P* = 0.0136), 4% increase in 10 th percentile proportion (*P* = 0.0257), and a 184% increase in 90 th percentile proportion (*P* = 0.0257). No statistically significant differences in high-order radiomic features were observed in R vs. PR/NR at PRT.

### PWI/DCE morphologic patterns, qualitative and semiquantitative parameters at PRT

**Fig. 3:**
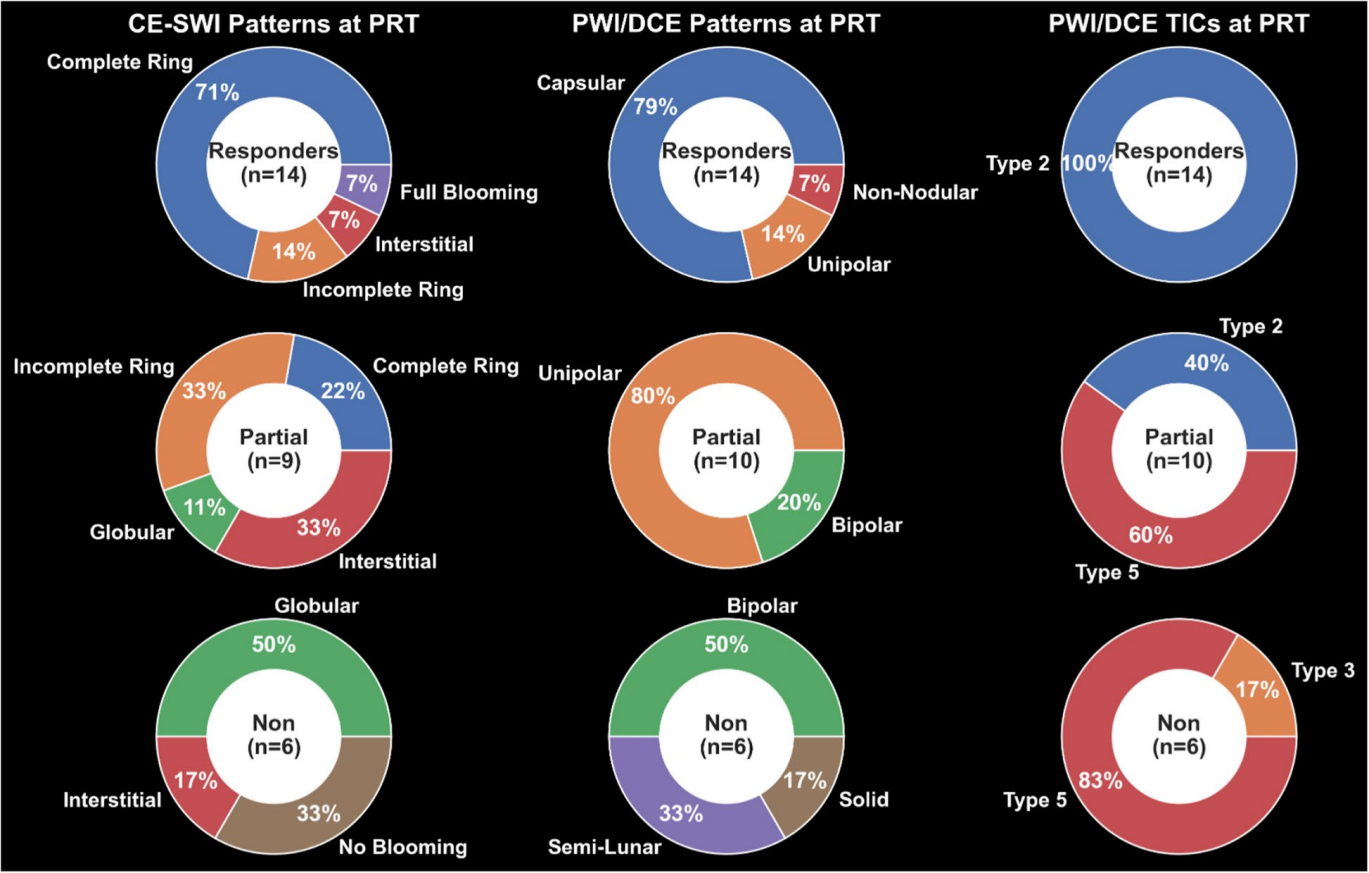
Frequency of CE-SWI morphological patterns, PWI/DCE morphological patterns, and PWI/DCE time-intensity curves (TICs) observed in responders, partial responders, and non-responders at post-radiation (PRT). Please refer to Tables 2–4 for examples of these patterns. CE-SWI Complete-Ring, PWI/DCE Capsular pattern, and PWI/DCE TIC type II are the most prevalent patterns observed in responders, while PWI/DCE Unipolar enhancement and PWI/DCE TIC type V are often seen in PR/NR

**Fig. 4:**
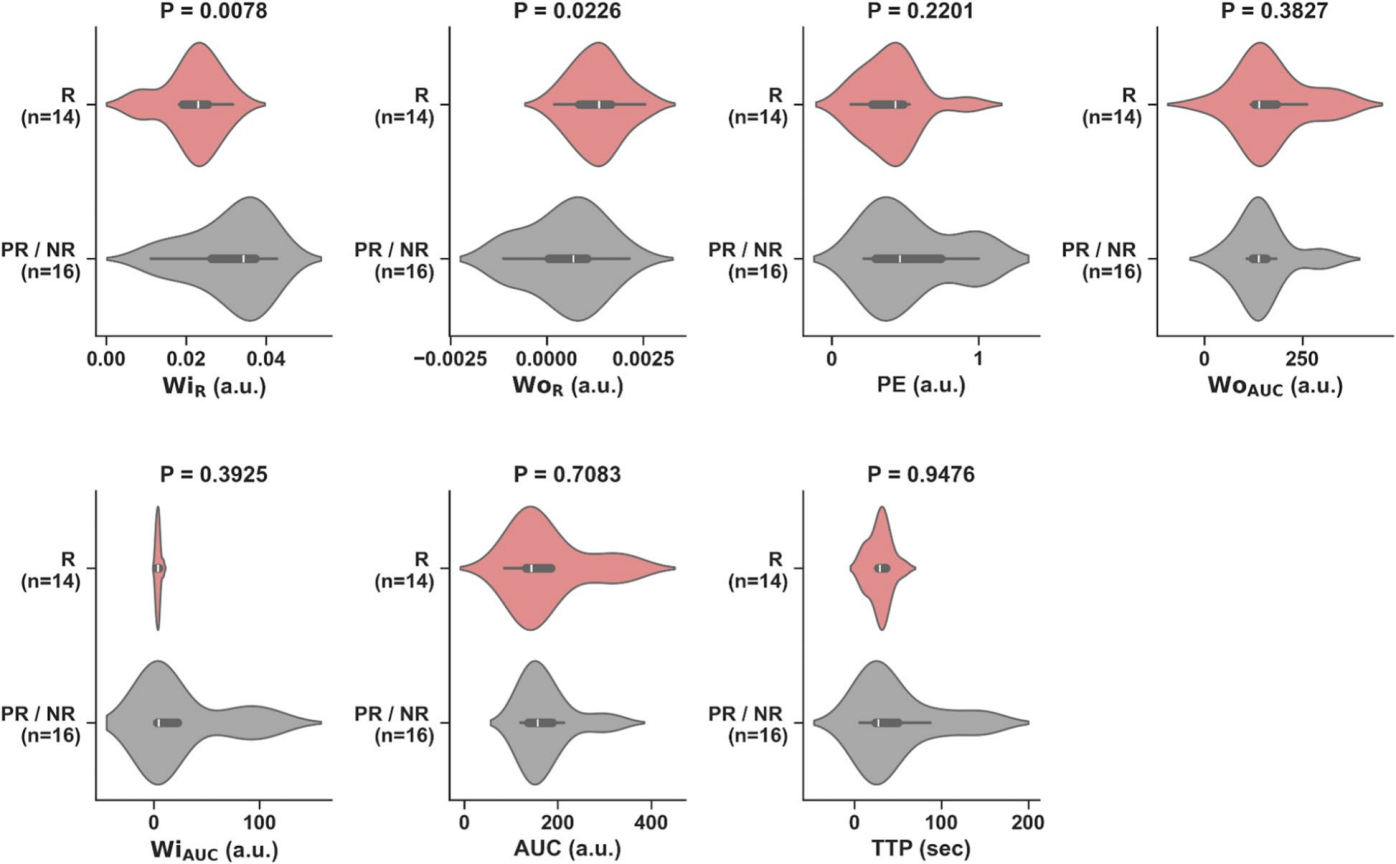
Violin plots of seven PWI/DCE semi-quantitative parameters comparing responders (R) vs. partial/non-responders (PR/NR) at post-radiation (PRT). Significant differences (*P* < 0.05) were observed in WiR and WoR. a.u.: Arbitrary Units

On PWI/DCE images, 79% of R displayed a Capsular pattern (*P* = 1.49 × 10^–7^), and 100% demonstrated a TIC-type II (*P* = 8.32 × 10^–7^) (Fig. 3). 80% of PR showed a Unipolar pattern (*P* = 1.03 × 10^–5^), 60% expressed a TIC-type V (*P* = 0.06). 50% of NR displayed a Bipolar pattern (*P* = 0.1562), and 83% expressed a TIC-type V (*P* = 0.0302). Statistical significance for wash-in rate (WiR; *P* = 0.0078) and wash-out rate (WoR; *P* = 0.023) was observed, separating responders vs. partial/non-responders (Fig. 4).

#### CE-SWI morphologic patterns and radiomics at PRT

**Fig. 5:**
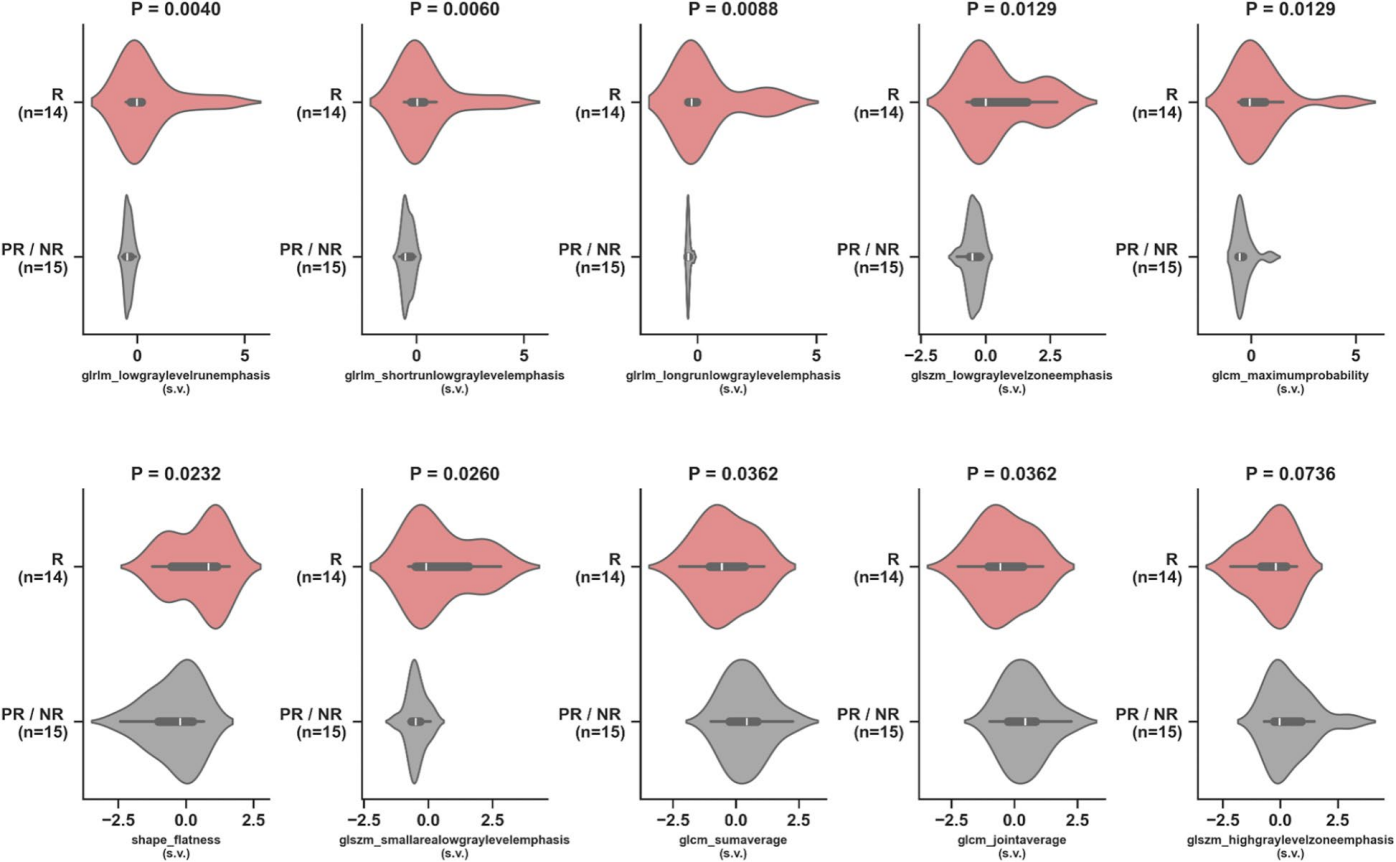
Violin plots of the top 10 CE-SWI radiomic features comparing responders (R) vs. partial/non-responders (PR/NR) at post-radiation (PRT). Statistically significant differences (*P* < 0.05) were observed in nine out of 10 radiomic features. s.v.: Standardized Value

A CE-SWI Complete Ring pattern was observed in 71% of R (*P* = 7.71 × 10^–6^), an Incomplete Ring pattern was observed in 33% of PR (*P* = 0.2751), and a Globular Pattern in 50% of NR (*P* = 0.1562) (Fig. 3). Post-radiation/Pre-operative CE-SWI derived GLRLM texture radiomic features, including GLRLM Low Gray Level Run Emphasis (*P* = 0.004), GLRLM Short Run Low Gray Level Emphasis (*P* = 0.006) and GLRLM Long Run Low Gray Level Emphasis (*P* = 0.0088) significantly discriminated R from PR/NR (Fig. 5).

#### ROC analysis of CE-SWI and PWI/DCE morphologic patterns and qualitative features

**Fig. 6:**
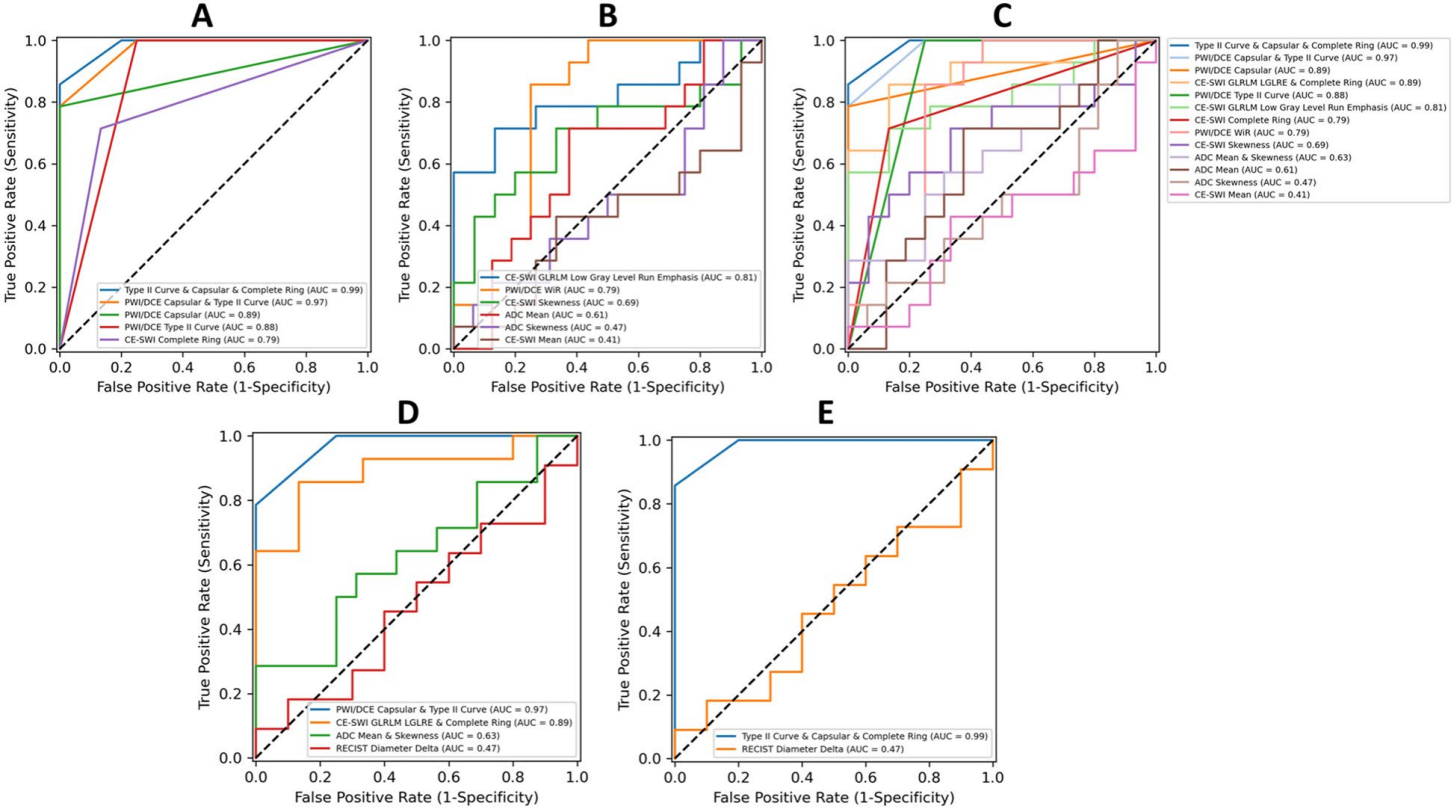
Receiver operating characteristic curves of classification models comparing responders vs. partial/non-responders at post-radiation based on (**A**) morphologic and qualitative features, (**B**) radiomic features, and (**C**) morphologic, qualitative, and radiomic features, (**D**) best performing DWI/ADC, PWI/DCE, and CE-SWI models, and (**E**) best-performing model overall. AUC: Area Under Curve

ROC analysis comparing R (*n* = 14) and PR/NR (*n* = 16) at PRT demonstrated that the model combining the PWI/DCE TIC-type II, PWI/DCE Capsular pattern and CE-SWI Complete Ring pattern yielded the highest classification performance (AUC = 0.99) (Fig. 6A), outperforming models based on PWI/DCE Capsular and TIC-type II (AUC = 0.97), PWI/DCE Capsular (AUC = 0.89), PWI/DCE TIC-type II (AUC = 0.88), and CE-SWI Complete Ring (AUC = 0.79).

#### ROC analysis of DWI/ADC and CE-SWI radiomic features

ROC analysis comparing the most significant radiomic features in R (*n* = 14) vs. PR/NR (*n* = 16) at PRT showed that the model based on CE-SWI GLRLM Low Gray Level Run Emphasis, which measures the concentration of low gray-level values in the image, yielded the best classification performance (AUC = 0.81) (Fig. 6B), outperforming models based on PWI/DCE WiR (AUC = 0.79), CE-SWI skewness (AUC = 0.69), ADC mean (AUC = 0.61), ADC skewness (AUC = 0.47), CE-SWI mean (AUC = 0.41) and RECIST (AUC = 0.47).

A comparison of the ROC curves of all morphologic, qualitative, and radiomic features is presented in Fig. 6C. In addition, a comparison of the best-performing models based on the most relevant features from DWI/ADC, PWI/DCE, and CE-SWI against RECIST is presented in Fig. 6D**.** Finally, Fig. 6E demonstrates that the model with the best performance combines the PWI/DCE TIC-type II, PWI/DCE Capsular pattern, and CE-SWI Complete Ring pattern (AUC = 0.99), significantly outperforming RECIST (AUC = 0.47).

## Discussion

### Treatment effect in UPS

After treatment, undifferentiated pleomorphic sarcoma (UPS) can show hemosiderin deposition (TIH), granulation tissue formation, fibrosis (TIF), and sometimes calcification. Evaluating the response of soft tissue sarcomas (STS) can be difficult after systemic or radiation therapy, as most treated STSs contain a mix of tissue components, including tumor cells, TIH, and TIF. Additionally, viable tumor cells may be replaced with benign tissue, such as fibrosis (TIF) and granulation tissue, rather than undergoing liquefaction or hemorrhagic necrosis, which challenges conventional imaging-based determination of the degree of treatment response. Conversely, the tumor may decrease, increase, or remain the same size. Response criteria such as RECIST require a significant reduction in tumor size to indicate a positive response. Our study results revealed pseudo-progression at PC and universal stability at PRT by RECIST in both responders and partial/non-responders. This suggests the unreliability of RECIST, WHO, and volumetric measurements for predicting histopathological effects, distinguishing between responders and partial/non-responders, and assessing overall treatment effectiveness.

### DWI/ADC features in UPS treatment assessment

Our results indicated that from BL to PRT, the ADC mean significantly increased by 35% in responders, consistent with previously reported findings [3, 18, 19]. This increase in ADC mean was accompanied by a significant reduction in skewness (− 136%) and a significant increase in the 90 th percentile proportion (+ 363%). In summary, the post-therapeutic first-order radiomic trends displayed by responders, including high ADC mean, low skewness, and high 90 th percentile proportion, were in agreement with the right-sided displacement of the ADC histogram typically observed in successfully treated UPS with > = 90% PATE [18, 19].

### PWI/DCE features in UPS treatment assessment

Our results have shown that a Capsular pattern is a typical PWI/DCE morphologic feature in the responder group (*P* = 1.49 × 10^–7^). A clinical radiologist can readily recognize this pattern without the need for post-processing software as is required for first- and high-order radiomic feature extraction. However, suboptimal responders tend to display a Unipolar or Bipolar pattern at PRT. The finding of the described patterns appears to follow an evolution where Bipolar seems to be a baseline default appearance of untreated or unsuccessfully treated tumors. Following a partial treatment effect, a unipolar pattern becomes prevalent. When successful therapy is completed, a capsular pattern emerges, indicating a natural sequence of events going from bipolar to capsular, rooted in the vascular organ-like anatomy of the tumor, where a dominant arterial pole and a non-dominant arterial opposite pole display maximum vascularity in opposition with a central/equatorial area of decreased perfusion and spontaneous necrosis and hemorrhage. In other words, the capsular pattern represents the complete ablation of dominant and non-dominant vascular poles. [24]. Our results also demonstrated that the TIC-type II displayed the strongest statistical association with response at PRT (*P* = 8.32 × 10^–7^). At the same time, TIC-types III, IV, and V were associated with ineffective treatment of UPS. The TIC universally transitions to type II following successful therapy with greater than 90% PATE. Therefore, after adequate treatment, there is significant resolution of the arterial hypervascularity previously represented as the rapid early uptake seen in TICs III, IV, and V, converting into a slow-ascending curve without the early rapid uptake characterized by TIC II. This transition is highly associated with successful therapy at PRT in responders. Finally, our findings demonstrated that both qualitative and semi-quantitative parameters, which represent a biomarker of arterial flow in active tumors, show the potential to distinguish between responders and partial/non-responders.

### CE-SWI features in UPS treatment assessment

The relatively recent and novel incorporation of CE-SWI in MSK oncologic imaging has helped to differentiate between benign and malignant soft tissue tumors, as high-grade tumors tend to have spontaneous central hemorrhage patterns [29]. In our institution, we have found that the CE-SWI sequence can demonstrate the viable enhancing portions of an STS separately from T2* hypointense hemorrhagic components (both of which can demonstrate high T1 signal), suggesting its utility as a helpful mechanism for evaluating treatment response. TIH is typically associated with low SWI-mean values and left-sided intensity histogram displacement with positive skewness. Our results have shown that a complete T2* hypointense ring is a typical CE-SWI morphologic pattern in the responder group [11, 21, 22, 29]. It is a finding readily recognized by the clinical radiologist without the need for post-processing software. The results observed within the high-order radiomic analysis of R vs. PR/NR highlight the value of the grey-level run length matrix (GLRLM). The GLRLM was introduced to define texture features and assess the distribution of discretized grey levels in an image or a stack of images. The GLRLM indicates a highly organized/concentrated pattern of T2* hypointense/low-intensity voxels representing the hemosiderin wall impregnation observed in responding UPS.

### Selection of the best UPS response model

#### Combining features from different sequences

By comparing the diagnostic performance of DWI/ADC, PWI/DCE, and CE-SWI derived morphologic, qualitative, semiquantitative, and radiomic features by ROC analysis, we observed that the model combining the pre-operative/PRT PWI/DCE TIC-type II, PWI/DCE Capsular and CE-SWI Complete Ring patterns yielded the highest area under the curve (AUC = 0.99) in separating R vs. PR/NR, demonstrating superiority over individual or combined models based on DWI/ADC, PWI/DCE and CE-SWI morphologic, qualitative, semiquantitative, and first- and high-order radiomics.

When analyzing models by sequence rather than by feature, Perfusion Imaging (PWI/DCE) using TIC-type II and Capsular pattern (AUC 0.97) and Contrast-Enhanced Susceptibility Imaging (CE-SWI) combining texture radiomics/GLRM and complete-ring (AUC 0.89) displayed the highest performance when compared with RECIST (AUC 0.47), nearly doubling its diagnostic performance.

Contrary to prior reports, DWI/ADC played a secondary role in predicting response. ADC mean (AUC = 0.61) and ADC mean & skewness (AUC = 0.63) showed superior performance compared with RECIST (AUC = 0.47), although inferior to PWI/DCE and CE-SWI when analyzed independently or combined. The above could, at least in part, be explained by the opposing effect of TIH, which tends to spuriously lower ADC values in responding tumors that otherwise could display a more significant increase in ADC. Quantifying the magnitude of such phenomena and their impact on ADC measurements is a subject for a separate and dedicated research analysis.

**Fig. 7:**
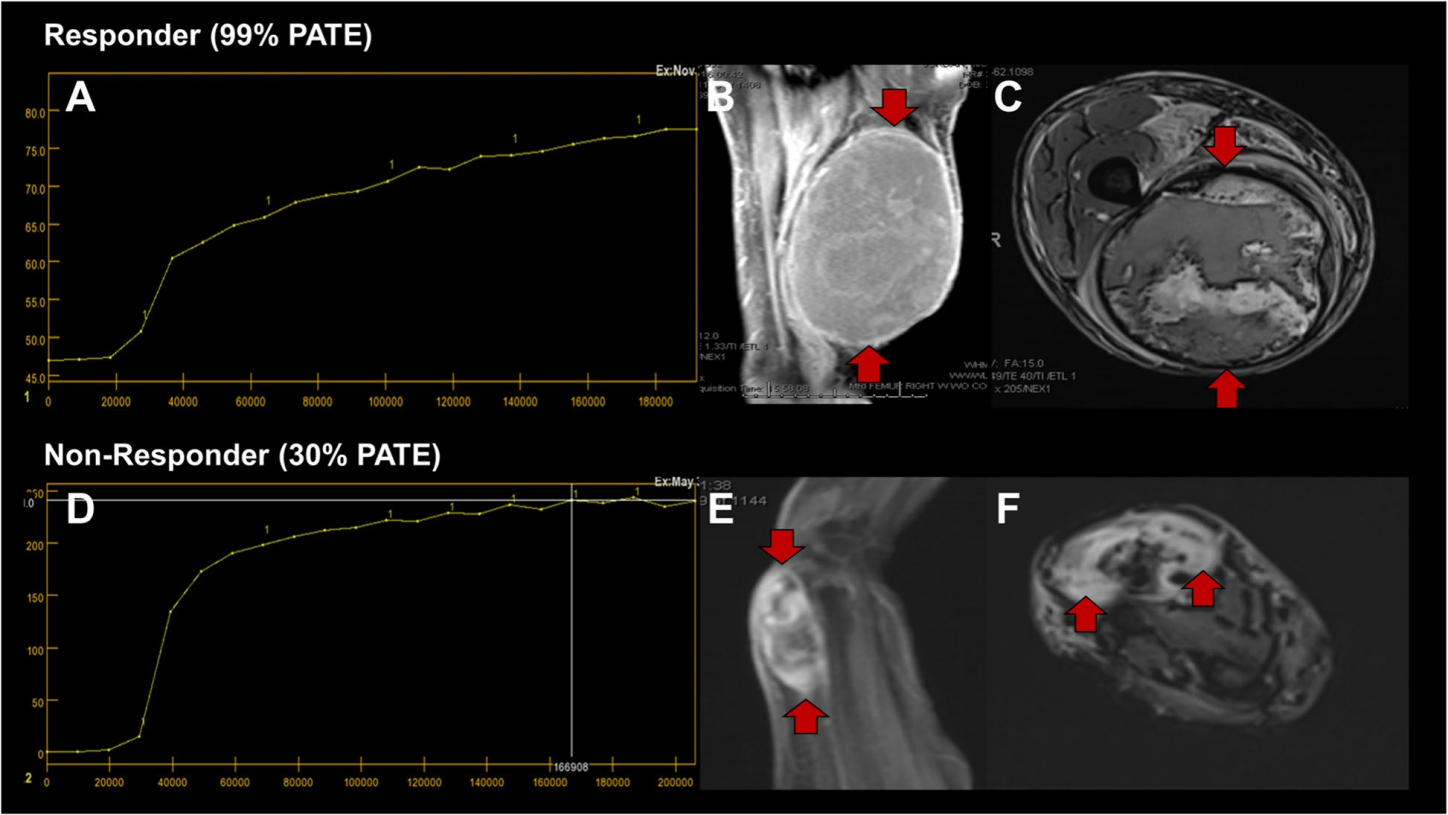
Top panels: Representative UPS responder displaying a PWI/DCE type II curve (**A**), PWI/DCE Capsular pattern (**B**), and CE-SWI complete ring pattern (**C**) at PRT. Bottom panels: Representative UPS non-responder displaying a PWI/DCE type V curve (**D**), PWI/DCE bipolar pattern (**E**), and CE-SWI globular pattern (**F**) at PRT. PATE: Pathology-Assessed Treatment Effect

Figure 7 displays a representative UPS responder case demonstrating a TIC-type II, PWI/DCE Capsular pattern, and CE-SWI Complete Ring pattern at PRT, compared to a representative UPS non-responder case. Response assessment based on the Capsular pattern, Complete Ring pattern, and TIC-type II provides a valuable multiparametric approach to routine clinical practice. These features can be identified subjectively and do not require post-processing software. Using mp-MRI, including DWI/ADC, PWI/DCE, and CE-SWI, demonstrates the potential for accurate treatment response assessment in UPS patients, significantly outperforming conventional metrics such as RECIST (Fig. 6). Institutions that have not yet developed CE-SWI can rely on a single-sequence approach using a perfusion-based model with a high diagnostic performance.

#### Study limitations

Our study presented the following limitations: 1) the sample size included in the analysis was relatively small (*n* = 33) and future studies with a significantly larger number of UPS patients will be needed to validate these promising results further; 2) performing mp-MRI in routine clinical practice is challenging as it typically requires a familiarized clinical radiologist and the implementation of the DWI/ADC, PWI/DCE, and CE-SWI sequences in the MRI protocol. However, in our experience, these sequences usually add no more than 15 min to the routine MRI exam while adding valuable functional information to the study. As a result, mp-MRI has been adopted as part of our current institutional routine extremity tumor MRI protocol, allowing clinicians and radiologists from our institution to become familiar with it over the last four years [3, 5]; 3) Our manual volumetric segmentation method could be prone to biases from inter- and intra-rater variability since it was performed by one research assistant in consultation with an experienced radiologist. Ongoing research efforts to overcome this limitation aim at developing a deep learning-based semi-automated lesion segmentation tool that can expedite this process and reduce potential biases in manual segmentation; 4) Finally, the relatively large number of radiomic features investigated in this study (107 features) added complexity and potential redundancy to our statistical analysis. Subsequent studies to improve our statistical methods will examine the use of mp-MRI-derived radiomics and morphologic features in machine learning models trained to assess UPS response automatically and objectively. Nevertheless, the proposed mp-MRI models demonstrated the potential to outperform conventional size-based metrics such as RECIST, WHO, and volumetric measurements in predicting treatment-induced histopathologic changes and overall treatment effectiveness in UPS.

## Conclusion

Mp-MRI-derived radiomic features can be used to establish predictive models for UPS treatment response assessment that outperform RECIST and other conventional size-based response assessment metrics. Observing a pre-operative/PRT PWI/DCE TIC-type II, PWI/DCE Capsular pattern and CE-SWI Complete Ring pattern have been shown to predict successfully treated UPS patients with > = 90% PATE. This offers a valuable and reliable approach primarily built on PWI/DCE and CE-SWI for practicing clinical radiologists without the need for complex post-processing or dedicated software. Contrary to prior reports, DWI/ADC played a secondary role in predicting treatment response in UPS. Although superior to RECIST, it underperformed compared to PWI/DCE and CE-SWI.

Although the mp-MRI approach provides the highest predictive performance when combining Perfusion (PWI/DCE) and Contrast-enhanced Susceptibility Imaging (CE-SWI), institutions that have not yet implemented the use of CE-SWI can rely on a single sequence approach based on PWI/DCE, combining the presence of TIC II and Capsular enhancement as criteria for response prediction.

## Data Availability

No datasets were generated or analysed during the current study.

## Abbreviations

ADC: Apparent diffusion coefficient
BL: Baseline
CE-SWI: Contrast-enhanced susceptibility-weighted imaging
DBMS: Database management system
DCE: Dynamic contrast enhancement
DWI: Diffusion-weighted imaging
Mp-MRI: Multiparametric magnetic resonance imaging
NR: Non-responders
PATE: Pathology-assessed treatment effect
PC: Post-chemotherapy
PD: Progressive disease
PR: Partial responders
PRT: Post-radiation therapy
PWI: Perfusion-weighted imaging
R: Responders
STS: Soft tissue sarcoma
SWI: Susceptibility-weighted imaging
TIF: Treatment-induced fibrosis
TIH: Treatment-induced hemorrhage
TIN: Treatment-induced necrosis
T2*: T2-star
UPS: Undifferentiated pleomorphic sarcoma
VOI: Volume of interest
RECIST: Response evaluation criteria in solid tumors
WHO: World health organization
